# Plague in Honolulu

**Published:** 1900-03-10

**Authors:** 


### PLAGUE IN HONOLULU.

REPORTS from Honolulu up to February 9th show that no new cases of plague had developed there since January 25th, and it was hoped that the outbreak had run its course. Let us hope so; unfortunately, it is a disease the usual course of which is to return again. There were still 7,400 Japanese and Chinese in quarantine. In dealing with the outbreak a large number of houses had been burned, 8,000 people having been rendered homeless by these proceedings. Attention was also paid to the destruction of the rats, a day having been set apart for a general raid on these pests. Every householder was supplied with rat poison to be used on the appointed day, and a reward was offered for all rats destroyed or caught.

